# The 1:1 co-crystal of triphen­yl(2,3,5,6-tetra­fluoro­benz­yl)phospho­nium bromide and 1,1,2,2-tetra­fluoro-1,2-di­iodo­ethane

**DOI:** 10.1107/S1600536813032522

**Published:** 2013-12-04

**Authors:** Gabriella Cavallo, Pierangelo Metrangolo, Franck Meyer, Tullio Pilati, Giuseppe Resnati, Giancarlo Terraneo

**Affiliations:** a NFMLab, Department of Chemistry, Materials and Chemical Engineering, "Giulio Natta", Politecnico di Milano, Via Mancinelli, 7, I-20131 Milano, Italy; b Laboratory of Biopolymers and Supramolecular Nanomaterials, Universitè Libre de Bruxelles (ULB), Campus de la Plaine, Boulevard du Triomphe, B-1050 Bruxelles, Belgium

## Abstract

The title compound, C_25_H_18_F_4_P^+^·Br^−^·C_2_F_4_I_2_, is a 1:1 co-crystal of triphen­yl(2,3,5,6-tetra­fluoro­benz­yl)phospho­nium (TTPB) bromide and 1,1,2,2-tetra­fluoro-1,2-di­iodo­ethane (TFDIE). The crystal structure consists of a framework of TTPB cations held together by C—H⋯Br inter­actions. In this framework, infinite channels along [100] are filled by TFDIE mol­ecules held together in infinite ribbons by short F⋯F [2.863 (2)–2.901 (2)Å] inter­actions. The structure contains halogen bonds (XB) and hydrogen bonds (HB) in the bromide coordination sphere. TFDIE functions as a monodentate XB donor as only one I atom is linked to the Br^−^ anion and forms a short and directional inter­action [I⋯Br^−^ 3.1798 (7) Å and C—I⋯Br^−^ 177.76 (5)°]. The coordination sphere of the bromide anion is completed by two short HBs of about 2.8 Å (for H⋯Br) with the acidic methyl­ene H atoms and two longer HBs of about 3.0 Å with H atoms of the phenyl rings. Surprisingly neither the second iodine atom of TFDIE nor the H atom on the tetra­fluoro­phenyl group make any short contacts.

## Related literature   

For a general discussion on halogen bonds (XBs) involving anionic halogen-bonding acceptors, see: for oxyanions, Abate *et al.* (2011 ▶); for chloride and bromide, Abate *et al.* (2009 ▶); for iodide, Metrangolo *et al.* (2008 ▶). For examples of reliable XB donors in an ionic context, see: Cavallo *et al.* (2010 ▶); Metrangolo *et al.* (2009 ▶); Logothetis *et al.* (2004 ▶). For different supra­molecular structures of halogen-bonded (poly)anions, see for: discrete adducts, Gattuso *et al.* (2007 ▶); infinite chains, Gattuso *et al.* (2006 ▶); comb-like arrays, Biella *et al.* (2009 ▶); ’ring and stick’ one-dimensional chains, Gattuso *et al.* (2009 ▶); two-dimensional layers showing Borromean inter­penetration, Liantonio *et al.* (2006 ▶). For very short XBs in the presence of HBs, see: Cametti *et al.* (2012 ▶); Gattuso *et al.* (2007 ▶). For a description of the Cambridge Structural Database, see: Allen (2002 ▶). For van der Waals radii, see Bondi (1964 ▶).
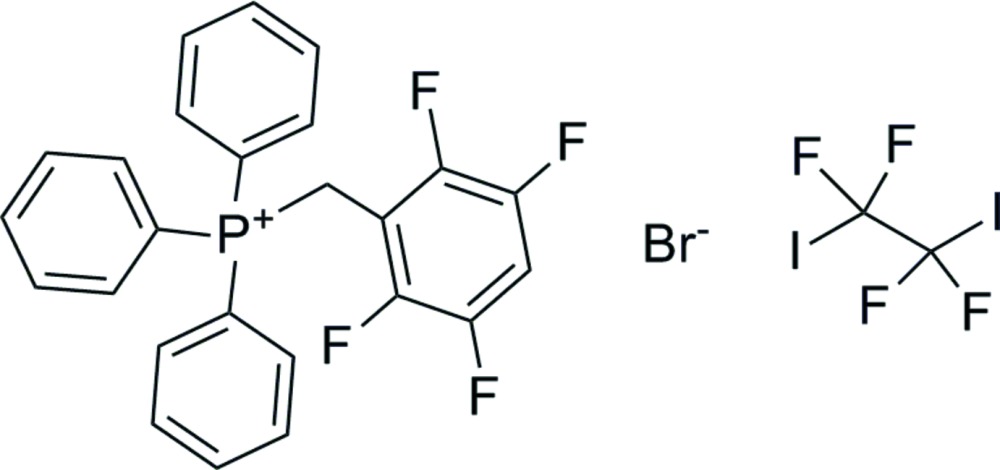


## Experimental   

### 

#### Crystal data  

C_25_H_18_F_4_P^+^·Br^−^·C_2_F_4_I_2_
*M* *_r_* = 859.09
Triclinic, 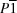

*a* = 9.6451 (10) Å
*b* = 10.9491 (12) Å
*c* = 13.8425 (15) Å
α = 78.07 (2)°
β = 79.08 (2)°
γ = 76.76 (2)°
*V* = 1376.7 (3) Å^3^
*Z* = 2
Mo *K*α radiation
μ = 3.87 mm^−1^
*T* = 90 K
0.26 × 0.14 × 0.10 mm

#### Data collection  

Bruker SMART APEX CCD diffractometer
Absorption correction: multi-scan (*SADABS*; Bruker, 2005 ▶) *T*_min_ = 0.795, *T*_max_ = 1.000
21807 measured reflections
10912 independent reflections
9550 reflections with *I* > 2σ(*I*)
*R*_int_ = 0.021

#### Refinement  

*R*[*F* ^2^ > 2σ(*F* ^2^)] = 0.030
*wR*(*F* ^2^) = 0.077
*S* = 1.04
10912 reflections
424 parameters
153 restraints
All H-atom parameters refined
Δρ_max_ = 1.33 e Å^−3^
Δρ_min_ = −0.82 e Å^−3^

### 

Data collection: *APEX2* (Bruker, 2005 ▶); cell refinement: *SAINT* (Bruker, 2005 ▶); data reduction: *SAINT*; program(s) used to solve structure: *SIR2002* (Burla *et al.*, 2003 ▶); program(s) used to refine structure: *SHELXL2012* (Sheldrick, 2008 ▶); molecular graphics: *ORTEP-3 for Windows* (Farrugia, 2012 ▶) and *Mercury* (Macrae *et al.*, 2006 ▶); software used to prepare material for publication: *SHELXL2012*.

## Supplementary Material

Crystal structure: contains datablock(s) global, I. DOI: 10.1107/S1600536813032522/qk2057sup1.cif

Structure factors: contains datablock(s) I. DOI: 10.1107/S1600536813032522/qk2057Isup2.hkl

Supporting information file. DOI: 10.1107/S1600536813032522/qk2057Isup3.cml

Additional supporting information:  crystallographic information; 3D view; checkCIF report

## Figures and Tables

**Table 1:** Hydrogen-bond geometry (Å, °)

*D*—H⋯*A*	*D*—H	H⋯*A*	*D*⋯*A*	*D*—H⋯*A*
C19—H19*A*⋯Br1	0.92 (2)	2.80 (2)	3.6866 (19)	163 (2)
C19—H19*B*⋯Br1^i^	0.92 (2)	2.83 (2)	3.7263 (19)	166 (2)
C16—H16⋯Br1^ii^	0.94 (1)	2.99 (2)	3.910 (2)	168 (2)
C11—H11⋯Br1^iii^	0.93 (1)	3.03 (2)	3.725 (2)	133 (2)

Symmetry codes: (i) 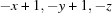; (ii) 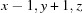; (iii) 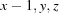.

**Fig. 1.**
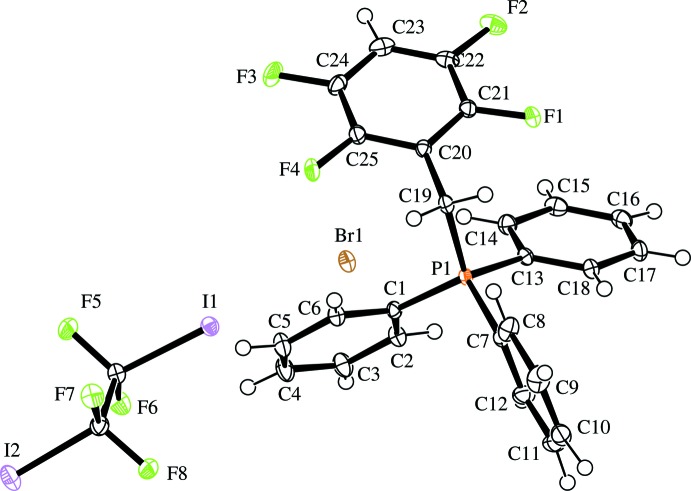
ORTEP view of the asymmetric unit of the title compound showing displacement ellipsoids at the 50% probability level.

**Fig. 2.**
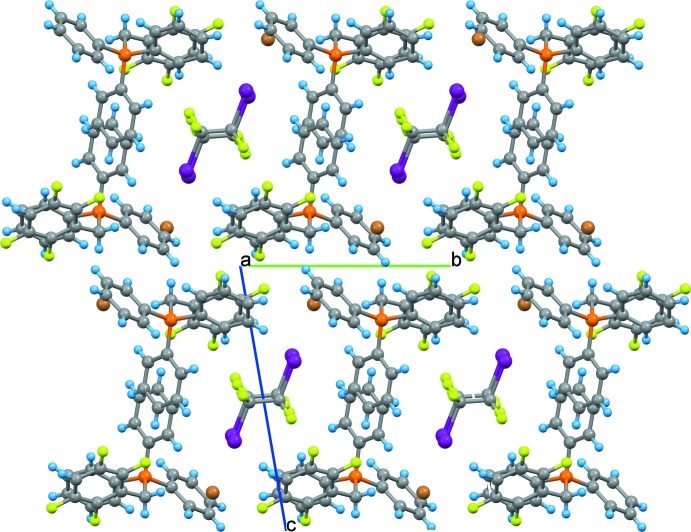
Mercury ball and stick plot of the complex viewed along a axis, showing the channels were the TFDIE molecules are segregated. Colour code: carbon, grey; hydrogen, light blue, fluorine, yellow; bromine, light brown and iodine, purple.

**Fig. 3.**
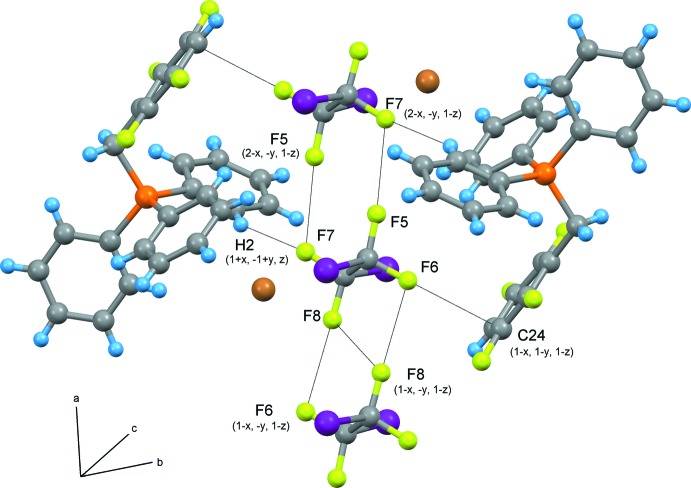
Mercury ball and stick plot of the complex, showing a part of the infinite chain of diiodotetrafluoro ethane molecules. Black dotted lines represent short intermolecular interactions. Colour code as Figure 2.

## supplementary crystallographic information

### 1. Comment

We have recently reported how oxyanions (Abate *et al.*, 2011) and chloride, bromide, (Abate *et al.*, 2009) or, more commonly, iodide anions (Metrangolo *et al.*, 2008) are effective acceptors of halogen bonds (XB) when interacting with a variety of XB donors, *e.g.* di- (Cavallo *et al.*, 2010) or tri-haloperfluorocarbons (Metrangolo *et al.*, 2009), haloimidazolium (Cametti *et al.*, 2012) or halopyridinium (Logothetis *et al.*, 2004) derivatives. In particular, naked halide anions were proven to work as particularly versatile XB acceptors and afforded supramolecular (poly)anions with quite different structures, *e.g.* discrete adducts (Gattuso *et al.*, 2007), infinite one-dimensional chains (Gattuso *et al.*, 2006), comb-like arrays (Biella *et al.*, 2009), 'ring and stick' one-dimensional chains (Gattuso *et al.*, 2009), and two-dimensional layers showing Borromean interpenetration (Liantonio *et al.*, 2006). In most of these structures, halide anions prefer to work as polydentate XB acceptors also when some H atoms in the cation could work as particularly effective hydrogen bond (HB) donor sites. In the present structure (Fig. 1), the bromide anion forms only one XB despite the composition of the system could allow for the bromide to function as a bidentate XB acceptor. Surprisingly, one iodine atom of TFDIE is not involved in any short contact. The positive phosphorus atom and the tetrafluorophenyl residue promote the acidity of the benzylic H atoms and they are both involved in short, probably strong, HBs with bromide anions, the coordination sphere of which is completed by two long, probably weak, HB interactions with H atoms of the non fluorinated phenyl rings (see Table A). A CSD search (CSD version 5.33; Allen (2002)) of C—I···Br^-^ interactions shows that the I···Br^-^ distances observed here (I···Br^-^ 3.1798 (7) Å) is below the lower quartile of the corresponding dataset (48 hits; mean I···Br^-^ distance: 3.399 Å; minimum I···Br^-^ distance: 3.093 Å), thus suggesting it is quite strong. The structural packing (Fig. 2) presents some interesting features. The tetrafluoro-1,2-diiodoethane molecules segregate in channels surrounded by cation molecules and do not show any rotational disorder. All the F atoms are engaged in F···F short contacts and produce an infinite ribbon (Fig. 3) which is anchored to the surrounding cations by H···F and C···F short contacts. Table B reports all the short contacts involving diiodoperfluoroethane molecules.

### 2. Experimental

In order to prepare the complex, a vial containing a CHCl_3_ solution of the two starting components (1:1 molar ratio) was sealed in a wide mouth vessel containg vaseline oil. Slow and isothermal CHCl_3_ diffusion furnished good quality crystals.

### 3. Refinement

All H atoms were located from difference Fourier maps and were then refined isotropically using a soft C—H distance restraint SADI 0.02 for all of them.

### Crystal data

**Table:**

C_25_H_18_F_4_P^+^·Br^−^·C_2_F_4_I_2_	*Z* = 2
*M**_r_* = 859.09	*F*(000) = 816
Triclinic, *P*1	*D*_x_ = 2.072 Mg m^−^^3^
*a* = 9.6451 (10) Å	Mo *K*α radiation, λ = 0.71073 Å
*b* = 10.9491 (12) Å	Cell parameters from 11010 reflections
*c* = 13.8425 (15) Å	θ = 2.3–34.3°
α = 78.07 (2)°	µ = 3.87 mm^−^^1^
β = 79.08 (2)°	*T* = 90 K
γ = 76.76 (2)°	Prism, colourless
*V* = 1376.7 (3) Å^3^	0.26 × 0.14 × 0.10 mm

### Data collection

**Table:**

Bruker SMART APEX CCD diffractometer	9550 reflections with *I* > 2σ(*I*)
ω and φ scans	*R*_int_ = 0.021
Absorption correction: multi-scan (*SADABS*; Bruker, 2005)	θ_max_ = 34.8°, θ_min_ = 1.9°
*T*_min_ = 0.795, *T*_max_ = 1.000	*h* = −15→15
21807 measured reflections	*k* = −17→17
10912 independent reflections	*l* = −21→21

### Refinement

**Table:**

Refinement on *F*^2^	Primary atom site location: structure-invariant direct methods
Least-squares matrix: full	Secondary atom site location: difference Fourier map
*R*[*F*^2^ > 2σ(*F*^2^)] = 0.030	Hydrogen site location: inferred from neighbouring sites
*wR*(*F*^2^) = 0.077	All H-atom parameters refined
*S* = 1.04	*w* = 1/[σ^2^(*F*_o_^2^) + (0.0439*P*)^2^ + 0.1396*P*] where *P* = (*F*_o_^2^ + 2*F*_c_^2^)/3
10912 reflections	(Δ/σ)_max_ = 0.002
424 parameters	Δρ_max_ = 1.33 e Å^−^^3^
153 restraints	Δρ_min_ = −0.82 e Å^−^^3^

### Special details

**Table:**

Experimental. OXFORD low temperature device.
Geometry. All e.s.d.'s (except the e.s.d. in the dihedral angle between two l.s. planes) are estimated using the full covariance matrix. The cell e.s.d.'s are taken into account individually in the estimation of e.s.d.'s in distances, angles and torsion angles; correlations between e.s.d.'s in cell parameters are only used when they are defined by crystal symmetry. An approximate (isotropic) treatment of cell e.s.d.'s is used for estimating e.s.d.'s involving l.s. planes.
Refinement. H atoms were refined by imposing soft restraint (all C—H distances nearly equal, see _iucr_refine_instructions_details)

### Fractional atomic coordinates and isotropic or equivalent isotropic displacement parameters (Å^2^)

**Table:**

	*x*	*y*	*z*	*U*_iso_*/*U*_eq_	
P1	0.22061 (5)	0.62628 (4)	0.20374 (3)	0.01039 (8)	
C1	0.23578 (19)	0.57972 (17)	0.33387 (13)	0.0118 (3)	
C2	0.1283 (2)	0.63023 (18)	0.40614 (14)	0.0158 (3)	
H2	0.054 (2)	0.6956 (19)	0.3883 (19)	0.024 (7)*	
C3	0.1375 (2)	0.5875 (2)	0.50599 (15)	0.0193 (4)	
H3	0.065 (2)	0.623 (2)	0.5528 (16)	0.020 (7)*	
C4	0.2531 (2)	0.4930 (2)	0.53479 (16)	0.0208 (4)	
H4	0.264 (3)	0.464 (3)	0.5999 (12)	0.036 (8)*	
C5	0.3586 (2)	0.4403 (2)	0.46351 (16)	0.0199 (4)	
H5	0.435 (2)	0.3789 (19)	0.4843 (19)	0.021 (7)*	
C6	0.3510 (2)	0.48281 (18)	0.36277 (15)	0.0158 (3)	
H6	0.420 (2)	0.446 (2)	0.3162 (14)	0.010 (5)*	
C7	0.1505 (2)	0.50892 (17)	0.16526 (14)	0.0134 (3)	
C8	0.2253 (2)	0.43955 (19)	0.09166 (16)	0.0194 (4)	
H8	0.3168 (17)	0.451 (2)	0.0619 (18)	0.019 (6)*	
C9	0.1600 (3)	0.3527 (2)	0.06445 (18)	0.0245 (4)	
H9	0.208 (3)	0.308 (2)	0.0141 (16)	0.029 (7)*	
C10	0.0227 (3)	0.3377 (2)	0.10923 (17)	0.0227 (4)	
H10	−0.029 (3)	0.287 (2)	0.090 (2)	0.029 (7)*	
C11	−0.0530 (2)	0.4085 (2)	0.18308 (17)	0.0225 (4)	
H11	−0.1430 (19)	0.393 (3)	0.215 (2)	0.035 (8)*	
C12	0.0113 (2)	0.4928 (2)	0.21135 (16)	0.0188 (4)	
H12	−0.043 (3)	0.542 (2)	0.2572 (16)	0.025 (7)*	
C13	0.09467 (19)	0.77405 (16)	0.18385 (13)	0.0116 (3)	
C14	0.0966 (2)	0.87510 (18)	0.23130 (15)	0.0157 (3)	
H14	0.156 (2)	0.867 (2)	0.2793 (15)	0.015 (6)*	
C15	0.0025 (2)	0.99060 (18)	0.20884 (16)	0.0170 (4)	
H15	−0.001 (3)	1.0570 (19)	0.2419 (18)	0.023 (7)*	
C16	−0.0912 (2)	1.00599 (18)	0.14058 (16)	0.0176 (4)	
H16	−0.161 (2)	1.0804 (18)	0.130 (2)	0.027 (7)*	
C17	−0.0919 (2)	0.90590 (19)	0.09336 (15)	0.0167 (4)	
H17	−0.151 (3)	0.917 (3)	0.0462 (18)	0.038 (8)*	
C18	0.0017 (2)	0.79030 (18)	0.11510 (14)	0.0140 (3)	
H18	0.001 (3)	0.726 (2)	0.081 (2)	0.033 (8)*	
C19	0.39365 (19)	0.64217 (17)	0.12875 (14)	0.0124 (3)	
H19A	0.452 (2)	0.5643 (16)	0.1463 (18)	0.019 (6)*	
H19B	0.374 (3)	0.657 (2)	0.0643 (12)	0.021 (7)*	
C20	0.44941 (18)	0.75023 (17)	0.14995 (13)	0.0117 (3)	
C21	0.40228 (19)	0.87529 (18)	0.10587 (14)	0.0138 (3)	
F1	0.31174 (12)	0.90025 (11)	0.03855 (9)	0.0172 (2)	
C22	0.4469 (2)	0.97554 (18)	0.12931 (15)	0.0170 (4)	
F2	0.39219 (14)	1.09432 (11)	0.08625 (10)	0.0231 (3)	
C23	0.5442 (2)	0.9558 (2)	0.19465 (16)	0.0195 (4)	
H23	0.577 (2)	1.0179 (18)	0.2150 (18)	0.015 (6)*	
C24	0.5952 (2)	0.8316 (2)	0.23629 (15)	0.0188 (4)	
F3	0.69239 (14)	0.80545 (14)	0.29958 (10)	0.0262 (3)	
C25	0.5492 (2)	0.73121 (18)	0.21507 (14)	0.0151 (3)	
F4	0.60278 (13)	0.61239 (11)	0.25752 (10)	0.0202 (2)	
I1	0.68637 (2)	0.17750 (2)	0.35932 (2)	0.01447 (3)	
C26	0.7339 (2)	0.07462 (18)	0.50522 (15)	0.0153 (3)	
F5	0.85788 (14)	0.09688 (12)	0.52343 (10)	0.0218 (3)	
F6	0.62843 (14)	0.11632 (12)	0.57682 (9)	0.0215 (3)	
C27	0.7486 (2)	−0.06934 (19)	0.51369 (15)	0.0163 (3)	
F7	0.86111 (13)	−0.11245 (12)	0.44721 (9)	0.0208 (2)	
F8	0.63051 (13)	−0.09436 (12)	0.49073 (10)	0.0203 (2)	
I2	0.78089 (2)	−0.17193 (2)	0.66122 (2)	0.02326 (4)	
Br1	0.61475 (2)	0.31789 (2)	0.14415 (2)	0.01496 (4)	

### Atomic displacement parameters (Å^2^)

**Table:**

	*U*^11^	*U*^22^	*U*^33^	*U*^12^	*U*^13^	*U*^23^
P1	0.01231 (18)	0.00947 (19)	0.00968 (19)	−0.00121 (14)	−0.00371 (15)	−0.00144 (15)
C1	0.0150 (7)	0.0117 (7)	0.0099 (7)	−0.0031 (6)	−0.0044 (6)	−0.0022 (6)
C2	0.0180 (8)	0.0153 (8)	0.0128 (8)	−0.0005 (6)	−0.0035 (6)	−0.0017 (6)
C3	0.0225 (9)	0.0212 (9)	0.0122 (8)	−0.0002 (7)	−0.0022 (7)	−0.0031 (7)
C4	0.0246 (9)	0.0262 (10)	0.0105 (8)	−0.0030 (8)	−0.0049 (7)	−0.0007 (7)
C5	0.0198 (9)	0.0208 (9)	0.0164 (9)	0.0015 (7)	−0.0064 (7)	0.0002 (7)
C6	0.0160 (8)	0.0152 (8)	0.0154 (8)	−0.0004 (6)	−0.0047 (6)	−0.0015 (7)
C7	0.0172 (8)	0.0111 (7)	0.0131 (8)	−0.0030 (6)	−0.0061 (6)	−0.0009 (6)
C8	0.0193 (9)	0.0180 (9)	0.0234 (10)	−0.0006 (7)	−0.0064 (7)	−0.0095 (8)
C9	0.0304 (11)	0.0173 (9)	0.0296 (12)	−0.0007 (8)	−0.0110 (9)	−0.0116 (8)
C10	0.0351 (11)	0.0161 (9)	0.0223 (10)	−0.0113 (8)	−0.0147 (9)	0.0006 (7)
C11	0.0286 (10)	0.0239 (10)	0.0193 (10)	−0.0164 (8)	−0.0057 (8)	0.0014 (8)
C12	0.0229 (9)	0.0200 (9)	0.0160 (9)	−0.0098 (7)	−0.0017 (7)	−0.0033 (7)
C13	0.0127 (7)	0.0096 (7)	0.0117 (8)	−0.0003 (6)	−0.0023 (6)	−0.0011 (6)
C14	0.0161 (8)	0.0136 (8)	0.0167 (9)	−0.0006 (6)	−0.0033 (7)	−0.0029 (7)
C15	0.0184 (8)	0.0115 (8)	0.0192 (9)	−0.0016 (6)	0.0012 (7)	−0.0036 (7)
C16	0.0140 (8)	0.0129 (8)	0.0210 (9)	0.0013 (6)	0.0005 (7)	0.0009 (7)
C17	0.0142 (8)	0.0174 (9)	0.0165 (9)	−0.0002 (6)	−0.0054 (7)	0.0009 (7)
C18	0.0142 (7)	0.0133 (8)	0.0134 (8)	−0.0003 (6)	−0.0040 (6)	−0.0011 (6)
C19	0.0149 (7)	0.0116 (7)	0.0104 (7)	−0.0011 (6)	−0.0039 (6)	−0.0015 (6)
C20	0.0113 (7)	0.0138 (8)	0.0099 (7)	−0.0019 (6)	−0.0015 (6)	−0.0022 (6)
C21	0.0121 (7)	0.0165 (8)	0.0117 (8)	−0.0024 (6)	−0.0011 (6)	−0.0008 (6)
F1	0.0170 (5)	0.0158 (5)	0.0172 (6)	−0.0012 (4)	−0.0066 (4)	0.0020 (4)
C22	0.0186 (8)	0.0122 (8)	0.0184 (9)	−0.0043 (6)	0.0027 (7)	−0.0024 (7)
F2	0.0276 (6)	0.0126 (5)	0.0264 (7)	−0.0040 (5)	0.0006 (5)	−0.0012 (5)
C23	0.0198 (9)	0.0219 (10)	0.0192 (9)	−0.0099 (7)	0.0036 (7)	−0.0082 (8)
C24	0.0171 (8)	0.0295 (11)	0.0132 (8)	−0.0098 (7)	−0.0034 (7)	−0.0047 (7)
F3	0.0260 (7)	0.0381 (8)	0.0212 (7)	−0.0136 (6)	−0.0116 (5)	−0.0045 (6)
C25	0.0146 (8)	0.0176 (8)	0.0125 (8)	−0.0028 (6)	−0.0031 (6)	−0.0002 (6)
F4	0.0214 (6)	0.0188 (6)	0.0208 (6)	−0.0026 (4)	−0.0123 (5)	0.0029 (5)
I1	0.01472 (6)	0.01475 (6)	0.01311 (6)	−0.00167 (4)	−0.00179 (4)	−0.00214 (4)
C26	0.0162 (8)	0.0151 (8)	0.0141 (8)	−0.0015 (6)	−0.0035 (6)	−0.0020 (6)
F5	0.0232 (6)	0.0217 (6)	0.0248 (7)	−0.0083 (5)	−0.0109 (5)	−0.0026 (5)
F6	0.0267 (6)	0.0208 (6)	0.0135 (6)	0.0010 (5)	0.0009 (5)	−0.0046 (5)
C27	0.0141 (8)	0.0183 (9)	0.0162 (9)	−0.0023 (6)	−0.0024 (6)	−0.0030 (7)
F7	0.0197 (6)	0.0216 (6)	0.0192 (6)	0.0011 (5)	−0.0004 (5)	−0.0071 (5)
F8	0.0206 (6)	0.0190 (6)	0.0241 (6)	−0.0073 (4)	−0.0088 (5)	−0.0010 (5)
I2	0.03227 (8)	0.01838 (7)	0.01800 (7)	−0.00200 (5)	−0.00925 (5)	0.00110 (5)
Br1	0.01754 (8)	0.01333 (8)	0.01289 (8)	0.00093 (6)	−0.00445 (6)	−0.00218 (6)

### Geometric parameters (Å, º)

**Table:**

P1—C7	1.7917 (19)	C14—H14	0.935 (14)
P1—C13	1.7920 (18)	C15—C16	1.388 (3)
P1—C1	1.7922 (19)	C15—H15	0.926 (14)
P1—C19	1.8153 (19)	C16—C17	1.389 (3)
C1—C2	1.398 (3)	C16—H16	0.936 (14)
C1—C6	1.405 (2)	C17—C18	1.389 (3)
C2—C3	1.377 (3)	C17—H17	0.920 (14)
C2—H2	0.920 (14)	C18—H18	0.930 (14)
C3—C4	1.394 (3)	C19—C20	1.507 (3)
C3—H3	0.932 (14)	C19—H19A	0.921 (13)
C4—C5	1.389 (3)	C19—H19B	0.920 (14)
C4—H4	0.909 (14)	C20—C21	1.389 (2)
C5—C6	1.386 (3)	C20—C25	1.394 (3)
C5—H5	0.924 (14)	C21—F1	1.341 (2)
C6—H6	0.919 (13)	C21—C22	1.383 (3)
C7—C8	1.388 (3)	C22—F2	1.345 (2)
C7—C12	1.406 (3)	C22—C23	1.376 (3)
C8—C9	1.399 (3)	C23—C24	1.378 (3)
C8—H8	0.925 (14)	C23—H23	0.922 (13)
C9—C10	1.382 (3)	C24—F3	1.346 (2)
C9—H9	0.924 (14)	C24—C25	1.378 (3)
C10—C11	1.402 (3)	C25—F4	1.340 (2)
C10—H10	0.934 (14)	I1—C26	2.175 (2)
C11—C12	1.375 (3)	C26—F6	1.349 (2)
C11—H11	0.931 (14)	C26—F5	1.351 (2)
C12—H12	0.921 (14)	C26—C27	1.532 (3)
C13—C18	1.386 (3)	C27—F8	1.338 (2)
C13—C14	1.404 (3)	C27—F7	1.347 (2)
C14—C15	1.393 (3)	C27—I2	2.159 (2)
			
C7—P1—C13	107.23 (9)	C16—C15—C14	120.78 (19)
C7—P1—C1	108.81 (9)	C16—C15—H15	119.1 (17)
C13—P1—C1	109.90 (9)	C14—C15—H15	120.0 (17)
C7—P1—C19	109.70 (9)	C15—C16—C17	120.20 (17)
C13—P1—C19	109.68 (9)	C15—C16—H16	120.9 (17)
C1—P1—C19	111.42 (9)	C17—C16—H16	118.6 (17)
C2—C1—C6	120.18 (17)	C16—C17—C18	119.52 (18)
C2—C1—P1	120.78 (14)	C16—C17—H17	120.2 (18)
C6—C1—P1	118.78 (14)	C18—C17—H17	120.3 (18)
C3—C2—C1	119.93 (17)	C13—C18—C17	120.53 (18)
C3—C2—H2	118.9 (17)	C13—C18—H18	121.8 (18)
C1—C2—H2	121.0 (17)	C17—C18—H18	117.7 (18)
C2—C3—C4	119.94 (18)	C20—C19—P1	111.81 (12)
C2—C3—H3	118.5 (16)	C20—C19—H19A	112.2 (16)
C4—C3—H3	121.6 (16)	P1—C19—H19A	103.6 (16)
C5—C4—C3	120.50 (19)	C20—C19—H19B	111.5 (16)
C5—C4—H4	117 (2)	P1—C19—H19B	103.4 (17)
C3—C4—H4	122 (2)	H19A—C19—H19B	114 (2)
C6—C5—C4	120.15 (18)	C21—C20—C25	116.27 (17)
C6—C5—H5	120.9 (16)	C21—C20—C19	121.35 (16)
C4—C5—H5	119.0 (16)	C25—C20—C19	122.37 (16)
C5—C6—C1	119.28 (18)	F1—C21—C22	118.80 (17)
C5—C6—H6	119.5 (15)	F1—C21—C20	119.61 (17)
C1—C6—H6	121.2 (15)	C22—C21—C20	121.59 (18)
C8—C7—C12	120.72 (18)	F2—C22—C23	120.42 (18)
C8—C7—P1	122.82 (15)	F2—C22—C21	118.00 (19)
C12—C7—P1	116.43 (14)	C23—C22—C21	121.58 (18)
C7—C8—C9	118.7 (2)	C22—C23—C24	117.24 (19)
C7—C8—H8	120.0 (15)	C22—C23—H23	126.3 (15)
C9—C8—H8	121.3 (15)	C24—C23—H23	116.5 (15)
C10—C9—C8	120.5 (2)	F3—C24—C25	118.05 (19)
C10—C9—H9	120.5 (18)	F3—C24—C23	120.27 (19)
C8—C9—H9	119.0 (18)	C25—C24—C23	121.68 (19)
C9—C10—C11	120.7 (2)	F4—C25—C24	119.11 (17)
C9—C10—H10	123.8 (17)	F4—C25—C20	119.30 (17)
C11—C10—H10	115.3 (17)	C24—C25—C20	121.58 (18)
C12—C11—C10	119.2 (2)	F6—C26—F5	107.16 (16)
C12—C11—H11	122.0 (17)	F6—C26—C27	109.03 (16)
C10—C11—H11	118.6 (17)	F5—C26—C27	108.36 (15)
C11—C12—C7	120.16 (19)	F6—C26—I1	109.67 (12)
C11—C12—H12	117.5 (17)	F5—C26—I1	110.37 (13)
C7—C12—H12	122.2 (17)	C27—C26—I1	112.11 (14)
C18—C13—C14	120.25 (16)	F8—C27—F7	107.53 (16)
C18—C13—P1	118.75 (14)	F8—C27—C26	109.93 (16)
C14—C13—P1	120.84 (14)	F7—C27—C26	108.98 (16)
C15—C14—C13	118.72 (18)	F8—C27—I2	108.64 (13)
C15—C14—H14	118.8 (15)	F7—C27—I2	108.96 (12)
C13—C14—H14	122.4 (15)	C26—C27—I2	112.67 (14)
			
C7—P1—C1—C2	−97.32 (17)	C15—C16—C17—C18	0.1 (3)
C13—P1—C1—C2	19.81 (18)	C14—C13—C18—C17	−0.9 (3)
C19—P1—C1—C2	141.60 (16)	P1—C13—C18—C17	−176.34 (15)
C7—P1—C1—C6	76.88 (17)	C16—C17—C18—C13	0.4 (3)
C13—P1—C1—C6	−165.98 (15)	C7—P1—C19—C20	173.43 (12)
C19—P1—C1—C6	−44.20 (18)	C13—P1—C19—C20	55.90 (15)
C6—C1—C2—C3	1.9 (3)	C1—P1—C19—C20	−66.02 (14)
P1—C1—C2—C3	175.99 (16)	P1—C19—C20—C21	−81.71 (19)
C1—C2—C3—C4	−0.7 (3)	P1—C19—C20—C25	97.62 (18)
C2—C3—C4—C5	−0.7 (3)	C25—C20—C21—F1	176.67 (16)
C3—C4—C5—C6	1.0 (3)	C19—C20—C21—F1	−4.0 (3)
C4—C5—C6—C1	0.2 (3)	C25—C20—C21—C22	−3.0 (3)
C2—C1—C6—C5	−1.6 (3)	C19—C20—C21—C22	176.32 (17)
P1—C1—C6—C5	−175.86 (16)	F1—C21—C22—F2	2.8 (3)
C13—P1—C7—C8	121.66 (17)	C20—C21—C22—F2	−177.53 (16)
C1—P1—C7—C8	−119.51 (17)	F1—C21—C22—C23	−177.31 (17)
C19—P1—C7—C8	2.62 (19)	C20—C21—C22—C23	2.4 (3)
C13—P1—C7—C12	−56.18 (17)	F2—C22—C23—C24	179.70 (17)
C1—P1—C7—C12	62.64 (17)	C21—C22—C23—C24	−0.2 (3)
C19—P1—C7—C12	−175.23 (14)	C22—C23—C24—F3	178.92 (18)
C12—C7—C8—C9	−0.3 (3)	C22—C23—C24—C25	−1.1 (3)
P1—C7—C8—C9	−178.07 (16)	F3—C24—C25—F4	−0.2 (3)
C7—C8—C9—C10	0.9 (3)	C23—C24—C25—F4	179.83 (18)
C8—C9—C10—C11	−0.5 (3)	F3—C24—C25—C20	−179.67 (17)
C9—C10—C11—C12	−0.5 (3)	C23—C24—C25—C20	0.4 (3)
C10—C11—C12—C7	1.1 (3)	C21—C20—C25—F4	−177.76 (16)
C8—C7—C12—C11	−0.7 (3)	C19—C20—C25—F4	2.9 (3)
P1—C7—C12—C11	177.23 (16)	C21—C20—C25—C24	1.7 (3)
C7—P1—C13—C18	−22.55 (18)	C19—C20—C25—C24	−177.67 (17)
C1—P1—C13—C18	−140.67 (15)	F6—C26—C27—F8	67.0 (2)
C19—P1—C13—C18	96.51 (16)	F5—C26—C27—F8	−176.66 (14)
C7—P1—C13—C14	162.02 (15)	I1—C26—C27—F8	−54.61 (18)
C1—P1—C13—C14	43.91 (18)	F6—C26—C27—F7	−175.36 (14)
C19—P1—C13—C14	−78.91 (17)	F5—C26—C27—F7	−59.0 (2)
C18—C13—C14—C15	0.8 (3)	I1—C26—C27—F7	63.01 (18)
P1—C13—C14—C15	176.16 (15)	F6—C26—C27—I2	−54.30 (18)
C13—C14—C15—C16	−0.3 (3)	F5—C26—C27—I2	62.02 (17)
C14—C15—C16—C17	−0.2 (3)	I1—C26—C27—I2	−175.94 (7)

### Hydrogen-bond geometry (Å, º)

**Table:**

*D*—H···*A*	*D*—H	H···*A*	*D*···*A*	*D*—H···*A*
C19—H19*A*···Br1	0.92 (2)	2.80 (2)	3.6866 (19)	163 (2)
C19—H19*B*···Br1^i^	0.92 (2)	2.83 (2)	3.7263 (19)	166 (2)
C16—H16···Br1^ii^	0.94 (1)	2.99 (2)	3.910 (2)	168 (2)
C11—H11···Br1^iii^	0.93 (1)	3.03 (2)	3.725 (2)	133 (2)

Symmetry codes: (i) −*x*+1, −*y*+1, −*z*; (ii) *x*−1, *y*+1, *z*; (iii) *x*−1, *y*, *z*.

### Table A. Short H···Br contacts.

**Table:**

	H···Br	C—H···Br
C19—H19A···Br1	2.797 (15)	163 (2)
C19—H19B···Br1^i^	2.826 (15)	167 (2)
C16—H16···Br1^ii^	2.991 (15)	168 (2)
C11—H11···Br1^iii^	3.025 (15)	133 (2)

Symmetry codes: (i) 1-x, 1-y, -z; (ii) 1+x, -1+y, z; (iii) 1+x, y, z.

### Table B. Contacts below the sum of van der Waals radii^a^ involving TFDIE.

**Table:**

	F···Z	C—F···Z
C26—F5···F7^i^	2.863 (2)	172.27 (12)
C26—F6···F8^ii^	2.901 (2)	102.90 (11)
C27—F7···F5^i^	2.863 (2)	117.40 (11)
C27—F8···F8^ii^	2.875 (3)	117.65 (12)
C27—F8···F8^ii^	2.875 (3)	117.65 (12)
C26—F6···C24^iii^	3.099 (3)	170.23 (12)
C27—F7···H2^iv^	2.62 (2)	148.9 (6)

Note: (*a*) van der Waals radii from Bondi (1964). Symmetry codes: (i) 2-x, -y, 1-z; (ii) 1-x, -y, 1-z; (iii) 1-x, 1-y, 1-z; (iv) 1+x, -1+y, z.
